# From Clinical Practice to the Classroom. Advantages and Disadvantages of Video and Paper Cases on the Motivation and Clinical Reasoning of Occupational Therapy Students

**DOI:** 10.3390/ijerph18189671

**Published:** 2021-09-14

**Authors:** María Rodríguez-Bailón, Ana Judit Fernández-Solano, Jose Antonio Merchán-Baeza, Laura Vidaña-Moya

**Affiliations:** 1 Department of Physiotherapy (Occupational Therapy), Faculty of Health Sciences, University of Málaga, 29071 Málaga, Spain; mariarbailon@uma.es; 2 Department of Occupational Therapy, School of Health Sciences, Catholic University of Murcia, 30107 Murcia, Spain; ajfernandez@ucam.edu; 3 Research Group on Methodology, Methods, Models and Outcomes of Health and Social Sciences (M3O), Department of Social Sciences and Welfare, Faculty of Health Science and Welfare, University of Vic-Central University of Catalonia (UVIC-UCC)), C/Sagrada Familia, 7, 08500 Vic, Spain; 4 Research Group GrEUIT, Escola Universitària d’Infermeria i Teràpia Ocupacional de Terrassa (EUIT), Universitat Autònoma de Barcelona, 08221 Terrassa, Spain; lauravidana@euit.fdsll.cat

**Keywords:** case-based learning, simulation, video cases, motivation, occupational therapy

## Abstract

Case-based learning enables the application of theory to practice using real-life patient cases. The present study aims to compare the effect between video cases and paper cases on motivation for learning and knowledge acquisition to perform a clinical reasoning case exercise by occupational therapy students. A mixed-methods design was used with 120 students randomized into two groups. All students conducted a clinical reasoning case exercise on the same case, although in different presentation formats: paper case and video case. The quantitative measures of this study were the scores of motivation for learning from the Instructional Material Motivation Survey and the grades of a clinical reasoning case exercise. The qualitative part was based on the positive and negative aspects perceived by the participants. The results showed that the motivation for learning was significantly higher for the video case compared to the paper case, although there were no differences in the grades of the clinical reasoning case exercise between the two groups. The video cases were perceived as more relevant to professional practice and more informative in terms of non-verbal communication and context. In light of the results, teachers could use these two formats of presentation of cases with different objectives.

## 1. Introduction

Case-based learning (CBL) involves the presentation of a well-designed clinical problem that enables students, through a real-life-based patient case, to apply theory to practice [1,2,3]. Extensive benefits of CBL have been reported in the health field [2,4,5], such as, for example, promoting active learning, with which students gain autonomy compared to a more passive traditional teaching approach, where information and knowledge are generated only by the teachers. In addition, this type of practice fosters a deep understanding of the subject among students, promoting long-term learning [6]. All this leads to CBL being effective at increasing student engagement and, therefore, motivation for learning, as is demonstrated by Raza, Qazi, and Umer, among others, through structural equation modeling [7,8].

One of the options to carry out CBL is making use of simulation through video case studies, standardized patients, or role-play [9]. Simulation is one of the learning modalities that has grown the most in the education of health professionals to prepare and contribute to the development of key competencies for clinical practice [10,11]. In various health disciplines, the use of video cases has been shown to increase students’ knowledge of their own professional role and their empathy [12], contributing to better preparation for their work in the clinical practice [13].

Both video cases and paper cases have advantages and disadvantages for teaching in higher education. Regarding the advantages, the cases presented on video have been shown to be perceived by students as more interesting and stimulating [14,15]. Thus, students seem to pay more attention with this learning method [16], which helps them to better remember long-term content [17]. In addition, the use of video cases has shown greater understanding of the professional role, more knowledge and understanding of the clinical aspects of the patient, greater ability to show empathy, and the feeling that they were useful for clinical practice (Bennett, Sylvia, Fitzgerald and Gibson, 2017). Another advantage is that this type of case presentation allows delving into and better capturing the psychosocial aspects of the person, beyond knowing and considering the biomedical data [18,19]. However, presented video cases may make it more difficult to identify important information [20], requiring more time compared to paper cases, due to the need to confirm the information presented [18]. Video cases can also hinder the possibility of revisiting the described information of the patients, thus reducing the reliability of the presented information [21].

Addressing the motivational factor to learn when examining the different modes of case presentation is essential, since the existence of a close relationship between motivation and effective learning has been evidenced [22]. The studies that explore the benefits of CBL on motivation have been conducted from different methodologies, based on different conceptualizations, and their results are contradictory. The qualitative study of Oosthuizen (2019), carried out with 22 speech therapy students, shows that, with the presentation of the case in video format, the students were able to imagine the clinical context more easily and better understand their profession, thus awakening in them a sense of purpose and motivation [23]. However, on the other hand, a cross-sectional study led by Ikegami obtained no significant differences between video and paper cases in student motivation for learning [18]. A review of the literature in this area shows the need to design new well-constructed studies, such as randomized studies, that analyze the effectiveness of both modalities on motivation [9].

In the same way, and in light of previous studies with no previous conceptual framework, it is essential to have a motivation model that articulates the evaluation of this important construct in order to know the effectiveness of the different presentation formats. In this sense, the ACRS (Attention, Confidence, Relevance, Satisfaction) model approach proposed by Keller (2010) could provide a structure from which to analyze motivation with practical application to instructional design [24]. This model breaks down motivation into four fundamental elements: attention, confidence, relevance, and satisfaction. It can help teachers to analyze the motivation of the group of students after the introduction of a new teaching material [25]. The element “attention” refers to how materials and methods capture the curiosity, enthusiasm, and interest of the students, making them adopt a participatory attitude towards learning due to their novelty. The ‘relevance’ or pertinence of the materials used refers to the connection that students feel towards materials and methods introduced in the learning process, with their learning needs, their objectives and past experiences, as well as with their learning preferences [26]. ‘Confidence’ is related to the feelings of personal control and the success expected to be attained by the end of the learning process following the methodology used. ‘Satisfaction’ is related to the positivity with which students face learning experiences. This dimension refers to the motivation of the student to keep learning, due to both extrinsic and intrinsic motivations.

Most studies that compare formats are based on student perceptions towards the advantages or difficulties evaluated by video cases and paper cases [15,17,19]. However, one of the objectives of health professionals’ training is to design tools and implement education strategies that lead to objectively improving effective clinical reasoning [27]. Measuring effective clinical reasoning is difficult, since it requires measurements of results that would involve the patients; however, there are some substitute evaluation instruments that could assess clinical reasoning in students. In this sense, the study by Murphy and Stav (2018) [28] compared the use of video cases and paper cases, and found that the former helped occupational therapy students to significantly improve their clinical reasoning, which was measured through the Health Science Reasoning Test [29]. However, clinical and professional reasoning should also involve training in specific cases of occupational therapy service users. In the discipline of occupational therapy, as in other health disciplines, it is essential to teach methods that promote professional reasoning, through the collection of personal and environmental information, with the aim of determining the occupational problems that arise, and their possible causes in order to establish the intervention goals to be achieved in each case [30]. Within clinical reasoning, diagnostic reasoning leads to the acquisition of clues about the components of performance based on a certain theoretical framework; this allows formulating the problem and synthesizing the gathered information [31]. According to Rogers and Holm [32], the diagnostic reasoning process involves gathering information on these four components: descriptive, explanatory, signs, and pathological. The descriptive component identifies the problems centered on occupation, while the explanatory component generates a hypothesis about the cause of that problem, which may be centered on the person or on the environment. The clues (signs) are the evidence, based on symptoms or problems reported by users or objective evidence, which allow recognizing occupational problems and their causes. The last component collects the medical or psychiatric information underlying the problem.

The main objective of this study was to analyze the benefits of presenting clinical cases in video format compared to paper format. Specifically, we evaluated the effects of both formats on the performance of a clinical reasoning case exercise and on the motivation of occupational therapy students, as well as the advantages and disadvantages of both formats perceived by students.

Our hypotheses suggest that the video format would be more motivating for students and could help them to obtain a better grade in the clinical reasoning case exercise.

## 2. Methods

### 2.1. Study Design

This mixed-methods study included a parallel-group, two-arm randomized controlled trial with 1:1 allocation through sealed envelopes and compared the effects of two clinical case presentation formats (video and paper) on the motivation for learning and knowledge acquisition to perform a clinical reasoning case exercise, exploring the advantages and disadvantages perceived by the participating students. The RCT addressed the question: In occupational therapy students, do video cases motivate and facilitate the realization of clinical reasoning case exercises to a greater extent compared to paper cases?

A mixed-methods design was selected, specifically an exploratory sequential design, with the aim of delving into the motivation/demotivation for learning. Moreover, this design allowed the students to express other aspects that facilitate (advantages) or hinder (disadvantages) the realization of the clinical reasoning case exercise based on the presentation format.

### 2.2. Participants

The study was conducted in four Spanish Universities. Each University randomized its participants into two groups: the experimental group and the control group. All participants were occupational therapy students who studied a subject related to the “Fundamentals of the Profession”. The study included students of the degree of occupational therapy who undertook subjects about the theoretical frameworks of occupational therapy (subjects taught in the first and second year). On the other hand, the study excluded those participants who did not attend the previous training sessions to perform the clinical reasoning case exercise.

In the participating universities, these subjects teach the fundamental theoretical concepts of occupational therapy, and students begin to develop competencies to know how to apply the different types of clinical reasoning. The study plans of the degree of occupation therapy in Spain last 4 years and are ruled by Order CIN/729/2009, of March 18, by the Spanish Ministry of Science and Innovation. The degree of occupational therapy is now beginning to be known in Spain among the general population and among potential university students, with over 50% of students registering for this degree as their first option.

### 2.3. Outcome Measures

Quantitative data were collected using two different instruments:

Instructional Material Motivation Survey (IMMS)

This is a 36-item questionnaire that evaluates four aspects of the motivational model proposed by Keller (attention, relevance, confidence, and satisfaction) [24]. Each of the items must be answered on a Likert scale with five response options, according to the degree of agreement, from total to partial scores, corresponding to each of the four elements that make up motivation. The “attention” subscale has 12 items, the “relevance” and “confidence” subscales have 9 items each, and the “satisfaction” subscale has 6 items. The preferred scoring method is to calculate the mean, dividing the total score for the subscale by the number of items on that scale. This converts the subscale scores to a number ranging from 1 to 5, facilitating comparisons between the subscales. The IMMS has been validated in previous studies and shows high internal consistency and reliability [33,34,35].

Grade of the Clinical Reasoning Case Exercise

To evaluate the clinical reasoning case exercise, an expert committee participated in the study. The committee was composed of eight occupational therapists (seven women and one man) each with over at least 10 years of clinical experience (physical and neurological rehabilitation, community intervention and mental health) and university teaching experience. Firstly, they were asked to resolve the clinical reasoning case exercise individually. Then, a meeting was held to discuss the differences between corrections and to generate a collaborative model of resolution. To respond to the descriptive component of the diagnostic reasoning process, an answer must be given at two levels: what occupation, and specifically what activity, are involved? Similarly, to address the explanatory component of the diagnostic reasoning process, different assessment levels are established based on: client factors, performance skills, performance patterns, context, and environment [32]. To identify these aspects, as the first process of clinical reasoning, it is necessary to establish a pre-selected theoretical framework [31]. To this end, we used the Occupational Therapy Practice Framework: Domain and Process [36]. This official document of the American Occupational Therapy Association aims to create a uniform terminology shared among all professionals, creating a summary of interrelated constructs that describe the practice of occupational therapy. Then, considering this model, a rubric was created by the four authors of this paper to correct the clinical reasoning case exercise. The rubric provided grades from 0 to 3 and asked four questions related to the knowledge acquired by the student: (1) Has the student prioritized the main activities? (2) Does the student use occupational therapy terminology from the AOTA framework? [36] (3) Does the student write the right content in each level? (4) Does the student justify the content of each level using the clues of the case? The grades were then converted to a scale of 0 to 10.

The clinical reasoning case exercise performed by the students was corrected by two members of the research team, who were blinded. To ensure inter-rater reliability, 20% of the diagnoses were corrected by both of them. Inter-rater reliability was assessed for 12 pairs of participants selected randomly from the sample, with an interclass correlation of 0.81. Once the inter-rater reliability was assessed, disagreements between the two coders were solved through discussion and re-assessment of tasks.

Advantages and Disadvantages Perceived in the Two Formats

Furthermore, a qualitative approach was used to measure the negative and positive aspects perceived by the participants of each of the conditions. To this end, two questions were asked: (1) What positive aspects did you identify while reading the report/watching the video that helped you to complete the clinical reasoning case exercise? This question referred to the description of facilitators/advantages perceived by the students that could help to complete the clinical reasoning case exercise; (2) what negative aspects did you identify while reading the paper case/watching the video case that made it difficult for you to complete the clinical reasoning case exercise? This question referred to the barriers detected by the students that could hinder the performance of the exercise and/or the disadvantages of this presentation format.

## 3. Procedure

During the period between October 2019 and January 2020, the four participating Universities, through four occupational therapy faculty members, conducted the intervention, which was divided into two sessions. The first one was a 2 h theoretical session on how to complete a clinical reasoning case exercise, using the Occupational Therapy Practice Framework (AOTA, 2008), responding to the descriptive and explanatory components and the corresponding indications. To this end, the students were taught to formulate seven levels (clinical reasoning case exercise). Levels 1–3 comprised the descriptive component that included information about the occupational area and activities involved. Levels 4–7 are related to the explicative component that includes the causes of the limitation on the occupational performance. This includes not only physical or cognitive factors related to the person but also the environment and all its dimensions (social, cultural, virtual, etc.).

In the second session, the students were randomly assigned to the experimental condition (video case) or the control condition (paper case). The students conducted the clinical reasoning case exercise in pairs, since the teaching plan of these subjects contemplates the development of the transversal competence of teamwork. They were given 45 min to either read the report or watch the video of the clinical case and complete the clinical reasoning case exercise. Although the form of presentation was different, the clinical case was related to the same patient and the information provided was the same for both formats of presentation. The case used for the study was that of a woman who had experienced a stroke and had aphasia and hemiparesis of the left half of her body. She was also diagnosed with major depression. The students previously received brief information about the medical diagnoses presented by the patient in the case. Information was provided on how she carried out her daily living activities at home. Her sister, who lived with her and was also her caregiver, was also interviewed. The conditions of both the social and physical environment that could impact their daily functioning were shown. The video case was performed by two professional actresses based on a script provided by an occupational therapist, who also assisted in directing functions. The video lasted 12 min and was hosted on YouTube for the students to have easy access.

For the completion of the clinical reasoning case exercise, they had to select the three activities that they would prioritize in the treatment and include information about the seven levels already mentioned. Once they had finished the clinical reasoning case exercise, they responded to the IMMS individually in a web version created by the authors.

After this phase, some of the students participating in the project (*n* = 73) participated in a cross-over design, that is, the participants performed a new case in the opposite format to the one they did in the first phase (the same pair was kept for the realization of this new exercise). Subsequently, these participants independently answered two open questions, through written answers, which they could access through a website created by the researchers. The narratives of the students who carried out the two conditions were analyzed, with the aim of incorporating the perceptions of those students who worked with both presentation formats. This purposeful sub-sample was constituted by students of different years of the degree of occupational therapy, in order to obtain greater representativeness of the total sample. Thus, quantitative and qualitative data were collected sequentially.

With the aim of making all the information available to students in one place, allowing them to revisit the case autonomously and carry out the exercise at their own pace, a web page was created to gather all the information needed to develop the sessions. It included the cases (video and paper), the required documents associated (e.g., the framework for occupational therapy practice), the IMMS, and the qualitative questions. The students completed the exercise and the evaluations in the classroom, where they received clarifications about the qualitative questions regarding the facilitators/advantages and barriers/disadvantages they had perceived. All the answers, both quantitative and qualitative, were anonymized. The students were asked to write a code in all the evaluations in order to link all the outcomes to each other (to carry out the partial correlations). To end the session, the clinical case was discussed in a large group (approximately 20 students) and all doubts were resolved.

## 4. Recruitment and Randomization

The occupational therapy students were informed in a face-to-face lesson about the study they were going to participate in at the four participating universities. Sealed enveloped randomization allocated the students equally (1:1) to video cases arm or paper cases arm. The researchers of each institution gave the sealed envelopes to the students, assigning them to both groups. To carry out the exercise, the students worked in pairs, which were randomized within each year and university. Pair randomization prevented the students from coming together by affinity.

Although blinding is nearly impossible in education research RCTs, the participants were not aware of the allocation sequence or their group allocation until the start of the intervention, and they did not know which case format was the experimental condition. In turn, the evaluators of the clinical reasoning case exercise were blinded with respect to the group to which each of them belonged.

## 5. Data Analysis

The quantitative and qualitative data were analyzed separately. The priority of the data analysis was placed on the quantitative data; the qualitative information allowed delving into and expanding the quantitative data. The integration of the data was carried out in the interpretation phase by all authors of this study.

### 5.1. Quantitative Measures

#### 5.1.1. Instructional Material Motivation Survey (IMMS)

With the aim of knowing the influence of the presentation format, an ANCOVA was carried out for each of the four dimensions of the IMMS (attention, relevance, confidence, and satisfaction). The covariate grade was included, since the sample was made up of students from different academic stages (mainly first and second year). The calculation of the scores for the four dimensions of the IMMS was carried out according to the manual proposed by Keller (2010) [24]. To this end, the mean of the corresponding items for each dimension was calculated.

#### 5.1.2. Grade of the Clinical Reasoning Case Exercise

Similarly, an ANCOVA was carried out to analyze the effect of the presentation format, (video or paper) on the grades obtained for the clinical reasoning case exercise. The year covariate was also included in the analysis. For both comparisons, the effect size was calculated with η2.

#### 5.1.3. Relationship between Motivation and the Grade of the Clinical Reasoning Case Exercise

An analysis of partial correlations between the total mean of the IMMS and the grades of the clinical reasoning case exercise was carried out, controlling for the academic stage separately for the students who carried out the clinical reasoning case exercise through video and those who carried it out by paper. The statistical analyses were carried out with JASP (0.8.6 Version) and IBM SPSS Statistics software (version 21).

### 5.2. Qualitative Analysis

Literally written responses were used for the independent analysis. The participants’ names were modified using an assigned code number in the transcripts and quotations. The analysis of the qualitative data was carried out through a thematic analysis. Three authors of this paper (MRB, AJFS, LVM) reviewed the written responses independently and generated initial codes that were meaningful (essence-capturing) units of analysis. Then, an open coding process was conducted, following a procedure of constant comparison [37]. The analysis conducted was inductive, and no pre-established categories were used before the coding process. Through a process of axial coding [38], the subcategories were integrated into the broader categories. Lastly, based on a process of selective coding, the categories were grouped by themes. Differences between researchers were resolved by discussion, consulting the literal answers when necessary. To facilitate the coding process, the text fragments were coded with ATLAS-Ti, a software for qualitative data analysis.

Trustworthiness was ensured by research triangulation, since three researchers analyzed the qualitative data, and also by methodological triangulation, due to the mixed methods design [39]. Although the sample was purposeful, data saturation was reached, since no new information was added throughout the analysis of the answers. The way in which the qualitative data were collected, i.e., through anonymously written answers, allowed the students to express their perceptions freely, without the influence of an academic context that could imply social desirability.

## 6. Ethical Considerations

This study follows the ethical standards of the Declaration of Helsinki (Manzini, 2000), obtaining a favorable ruling of the Ethical Committee for Experimentation of the University of Malaga (CEUMA 63-2019) and guaranteeing compliance with the Spanish law of protection of personal data (Organic Law 3/2018, of December 5). All participants received the informed consent form and were asked to sign it before being included in the study. Participation in this study was voluntary and did not imply compensation of any kind or impact on grades or academic record.

## 7. Results

### 7.1. Quantitative Results

IMMS

A total of 120 students completed the IMMS (107 women and 13 men). The mean age was 21.51 (SD: 4.77) years. Regarding the academic year, 78, 38, 3, and 1 participants were 1st-, 2nd-, 3rd-, and 4th-year students. A total of 62.5% of the students chose the degree of occupational therapy as their first option. With respect to the presentation mode, 56 students performed the exercise through a written report and 64 through the video of the case. Item 36 of the IMMS regarding the feedback obtained after performing the exercise could not be completed by some students from a university due to technical difficulties (five students who did the task through the report and eight students who did it through video). Therefore, the mean was calculated for the satisfaction dimension discarding the score from these students for this item.

A significant difference between the two formats was obtained in all dimensions of the IMMS (attention: F = 25.93, *p* < 0.001, η^2^ = 0.180; relevance: F = 7.49, *p* = 0.007, η^2^ = 0.060; confidence: F = 11.72, *p* < 0.001, η^2^ = 0.090), except for the satisfaction component (F = 3.34, *p* = 0.07, η^2^ = 0.027), obtaining higher scores in the students who complete the clinical reasoning case exercise through video. Differences in attention, relevance and confidence remained significant, even after adding the year covariate.

Table 1 shows the score of the dimensions of the IMMS according to the format.

#### 7.1.1. Grades of the Clinical Reasoning Case Exercise

No significant differences were obtained between the two formats of presentation of the clinical reasoning case exercise for their grade (F = 0.007, *p* = 0.934, η^2^ = 0.000) (see Table 1 for means and standard deviations). However, a year effect was found (F = 7.691, *p* = 0.006, η^2^ = 0.062). The second-year students scored significantly higher (mean = 5.84, SD = 1.86) than the first-year students (mean = 4.55, SD = 1.92).

#### 7.1.2. Relationship between Motivation and the Grade of the Clinical Reasoning Case Exercise

The relationship between motivation and the grades of the clinical reasoning case exercise were not significant neither in the students who carried out the exercise through video (r = 0.10; *p* = 0.44) nor in those who did it through the written report (r = −0.28; *p* = 0.10).

### 7.2. Qualitative Results

A total of 73 students completed the questionnaire. Three main themes were identified related to both formats of presentation of the clinical case: (a) aspects related to the format of presentation, (b) motivation to diagnose the case, (c) professional abilities acquired.

The themes and categories are presented in Table 2. In order to protect the privacy of the participants, pseudonyms were allocated to each of them.

#### 7.2.1. Aspects Related to the Format of Presentation

Video

The participants who received the information visually highlighted that, when watching the video, they could appreciate the patient’s occupational performance in a more realistic way, both her limitations and her abilities:

*It is much more visual, which gives us the chance to be more objective, because we can directly see how the person performs certain activities of daily living (st 89).*

*You really see the person and you can get an idea of how the illness is affecting her (st 67).*

Furthermore, watching the video gave the participants access to information that they considered important, such as non-verbal communication. Seeing her gesture, reactions, and movements and listening to her voice were some of the aspects mentioned by the participants:

*Thanks to non-verbal communication and people’s gestures you can have much more information that is sometimes missing in a written document (st 72). Because patient’s expressions show much more than words (st 110).*

The participants also mentioned that watching the case allowed them to better appreciate aspects related to the environment of the patient. These included both her physical context (including architectural barriers and accessibility limitations) and her social context (relationship with others and family opinions):

*You can check which limitations are more significant for the patient, thus getting a better idea of her physical environment (st 90).*

*You can observe the patient’s real situation and perceive her relationship with others (st 120).*

As a negative aspect of the video presentation, they highlighted that the information was not very specific and difficult to find, thus some important details for the clinical reasoning case exercise could be missed, which makes it more difficult to go back and get that information:

*I think that, when doing the case through the video, it is more difficult to notice the details, because a lot more attention is required. In the report it is already written (st 104).*

*I think […] the process of doing the clinical reasoning case exercise is slower, because you have to draw your own conclusions and perhaps there are a lot of details that you miss. Probably we will watch the video more than once to make a good clinical reasoning case exercise (St 99).*

Paper

On the other hand, the participants who were given the paper clinical case highlighted that the information was accessible, thus they had all the patients’ information always at hand and it was quicker for them to find what they considered important:

*You always have the information at hand, and it is much quicker to select the different aspects that you need to complete the task (st 13).*

*[…] you can check the information easily if you don’t remember something (st 60).*

They also talked about how clearly presented the information was, with detailed explanations, which made it easier to identify the occupational limitations of the patient:

*It is easier because everything is written, well explained and developed, so you do not have to think so much about the different details (st 28).*

*You can directly read the problems of the patient for whom we are conducting the clinical reasoning case exercise (st 40).*

As a negative aspect, most of the participants mentioned that they obtained less information, as it was difficult to imagine some occupational aspects when the patient could not be observed visually. Moreover, they stated that they missed some information, such as non-verbal communication, which makes the assessment more subjective:

*You don’t really see the person. I can’t treat a person only by reading a report. Talking to the person or seeing how she acts is very important (st 2).*

*You miss a lot of details when you don’t see her performance, including the way she communicates (st 26).*

#### 7.2.2. Motivation to Learn from the Case

Video

After the intervention, the participants identified some key points related to motivation.

Those who did the video case expressed that it was more enjoyable:

*It was entertaining; more interesting than reading a report (st 95).*

*It is more enjoyable, and you can observe things that do not appear at reports, such as facial expression (st 86).*

Paper

Those who did the paper case expressed that it was boring, since all the information was presented in the same way and was not appealing. This made the participants get distracted while reading the case and did not obtain all the information:

*It is boring and you do not connect so much with the person because it is more superficial (st 12).*

*You can get distracted while reading because it is not so enjoyable, and you can miss some important details (st 56).*

#### 7.2.3. Professional Abilities Acquired

Video

All participants reported that they learned some aspects that are important for their professional lives as occupational therapists.

Those who watched the clinical case highlighted that it gave them the chance to learn about the behavior of the professional:

*You get involved in the role of the therapist and see with your own eyes what the client’s behavior is like (st 88).*

*We put ourselves in the role of OTs and start a little bit in the world of a therapist, seeing real cases (st 119).*

They also mentioned that seeing the person doing the activities of daily living in such an explicit way, while also hearing her voice, made them feel more connected to the person’s problems and be more empathetic:

*Seeing the body expressions and movements and listening to her voice makes it easier to be empathetic with the patient and her situation…and it helps to establish a better occupational diagnosis (st 118).*

*It helps to connect more with the person and her problem […] (st 90).*

Paper

In contrast, those who read the case mentioned that it was difficult to imagine what the person was going through and how the situation was affecting her, making them less empathetic, even to the extent of not feeling in a position to treat someone only by reading a report:

*It’s difficult to put yourself in the patient’s shoes, because you’re not seeing her body expressions and/or evolution at each moment or activity that she is performing (st 27).*

*You don’t really see the person. I cannot treat a person just by reading a report. Seeing the person or talking to her is very important (st 6).*

## 8. Discussion

The aim of the present study was to explore the effect of the presentation format of a case, i.e., video or paper, on the motivation for learning and academic performance of occupational therapy students to complete a clinical reasoning case exercise in the first year of their university degree. The results showed that the motivation for learning was significantly higher for the video cases compared to the paper cases in three of the scales of the motivation model with practical application proposed by Keller (attention, relevance, and confidence), although there was marginal significance for the satisfaction scale. In this sense, to the best of our knowledge, this is the first mixed-methods study to include a randomized trial and demonstrate how the completion of a CBL task through video observation may be more motivating for higher education students than a paper case.

### 8.1. Attention

Regarding attention, the qualitative information corroborated the results of the IMMS, which indicated that the students who carried out the video case obtained higher scores in this motivational dimension. In this sense, the qualitative information revealed that the video case was more enjoyable and interesting, which is in line with results from similar studies [14,15]. This greater interest may be partially due, as one of the students commented, to the fact that aspects that do not appear in the reports can be observed in the video. The students emphasized that aspects of non-verbal communication are perceived in the video cases, as was reported by Nunohara et al. (2020) and Ikegami et al. (2017) with respect to psychosocial aspects [18,19]. This information is considered crucial to understand the person and put oneself in their shoes, thus fostering empathy, which has also been shown by several studies in the field of healthcare [19]. In this study, in addition, the possibility of observing the patient’s context became especially important, since it significantly impacts occupational performance and allows the students to directly appreciate performance without the interpretation that third parties must make when writing a report. This information is essential in clinical (diagnostic) reasoning, since this complex cognitive process seeks to move away from medical reductionisms through which the problems that people present are explained exclusively by organic causes [32].

### 8.2. Relevance

The qualitative results expand the data collected quantitatively on relevance, such as the relevance of the format to connect these contents to the students’ preferences, learning objectives, etc. The students highlighted empathy as an advantage of the video case, as well as the fact of making a more authentic and real image of the person, which helped them, to a greater extent, to approach the professional role that they will develop in the near future. In this sense, as was shown by Oosthuizen (2019) [23], a better understanding of the profession, especially in socio-health disciplines, generates more motivation among students.

### 8.3. Confidence

The students who completed the clinical reasoning case exercise in the video format also perceived greater personal control (understood as the subjective perception of attaining a personal goal) and more confidence in successfully passing future tests that include carrying out a similar exercise. This construct, also called performance self-efficacy (the student’s perception of his/her ability to safely achieve a good grade) [40], together with other more general variables, is a crucial indicator of academic performance [40].

### 8.4. Satisfaction

Although higher means were obtained for the students who completed the exercise through video, satisfaction, i.e., the positivity of the students in their learning experience, was not significantly different between the presentation formats. This may be due to the fact that video cases, although their advantages stand out, have also proven to have disadvantages, as indicated by the qualitative information. In line with the observations of other researchers [20,41], the participants stated that, in video cases, compared to paper cases, the information is more difficult to find and it is not as concrete, specific, and relevant to carry out the clinical reasoning case exercise, which generates greater confusion in the student. The students argued that, in the paper case, the information is more accessible, in line with Woodham et al. [20], showing that video cases hinder the work of critically reviewing the information presented.

### 8.5. Grades of the Clinical Reasoning Case Exercise

Motivation is key for meaningful learning; thus, it is important to know the effects of different formats of the learning materials in order to increase motivation and, therefore, facilitate involvement and learning.

Although there was a significant impact of the video case on student motivation, contrary to our hypotheses, this did not result in higher grades of the clinical reasoning case exercise, and no significant correlations were obtained between students for the IMMS results and grades of the clinical reasoning case exercise based on the presentation format.

This may be due to several reasons. Firstly, what the IMMS assesses is motivation with practical application for instructional design, that is, the creation of a learning environment and materials to enable the student to carry out certain tasks [42], such as performing a clinical reasoning case exercise. Considering the grades of the clinical reasoning case exercise as a measure of academic performance, we observe that there are many other individual factors that influence student performance that were not explored in this study, such as self-assigned minimal goal standards and academic self-efficacy [40]. The latter refers to general perceptions of academic ability, including the control that students perceive to have over their academic performance in their different years.

Secondly, perhaps the greatest impact of the different case presentation formats is not on procedural reasoning, where the student tries to identify the greatest number of causes of the alterations in the occupational performance, but on interactive reasoning [43,44,45]. This type of reasoning allows students and future occupational therapists to capture the experience of the person with occupational dysfunction, which in the clinical context translates into an increase in the active participation of the person and in the consolidation of the therapeutic relationship [43,44,45]. Therefore, in future research, it would be interesting to incorporate measures of the different types of reasoning that students develop when completing clinical reasoning case exercises.

### 8.6. Limitations

Finally, we must highlight some limitations of the study that could also explain these data. The tool to evaluate clinical reasoning was an instrument created ad hoc for this study and specifically for the discipline of occupational therapy, since no standardized tools have been developed to date for this purpose. This instrument was not validated in the study itself, although it followed a rigorous process in which experts participated in solving cases, and inter-observer reliability was calculated in the grades of the clinical reasoning case exercise.

Although collaborative work (e.g., working in pairs) is a common and recommended practice within the university context, which allows developing teamwork competencies, and also helps in the teaching–learning process [46], it is important to note that this fact, in this study, could have influenced the results. The motivation and qualification of the exercise, as well as the perception of the advantages and disadvantages of each of the formats, could be influenced by the other member of the pair. However, the design of the present randomized study, which has a large sample and equality in the conditions for carrying out the exercise, greatly mitigates this possible limitation.

## 9. Conclusions

The results of this mixed-methods study, which includes a randomized trial, show that students experience greater motivation with the practical application of an instructional design when carrying out a clinical reasoning case exercise in video format compared to a paper report. The video cases are perceived by students as more entertaining, more aimed at professional practice, and more informative in terms of non-verbal communication and the context in which the person develops. However, and in contrast to paper cases, in this video case, the information has been perceived as less structured, and it is difficult to find the most relevant data to complete the clinical reasoning case exercise. Nevertheless, it is important to mention that this perception may be due to this particular video case. Future designs with more structured scripts for video cases could help students in this regard. Regarding the grades of the clinical reasoning case exercise, no differences were found between formats or the relationship between this measure and the four motivational dimensions, which may be due to the more procedural and less interactive type of reasoning necessary to carry out this type of exercise.

### Practical Applications

These results highlight the effort made by faculty members to create and edit videos of simulated or real patients to teach their lessons and thus increase the motivation of the students. However, it is important to note that, depending on the teaching objectives pursued (whether they are focused on motivation or on supporting/structuring the information gathering process or the case data interpretation), the presentation could only be recommended in video format when the student is requested to identify relevant information, or a combination of both presentation formats to aid in the clue acquisition stage.

## Figures and Tables

**Table 1:** Means and standard deviations for each dimension of the IMMS and grades of the clinical reasoning case exercise according to the format (video or paper).

	Instructional Material Motivation Survey				Grades of the Clinical Reasoning Case Exercise
	Attention *	Relevance *	Confidence *	Satisfaction	Rating
Video	4.29 (0.59)	4.07 (0.53)	3.90 (0.49)	4.09 (0.72)	5.01 (1.84)
Paper	3.75 (0.56)	3.81 (0.49)	3.59 (0.48)	3.87 (0.60)	4.95 (2.10)

Note: * = *p* < 0.05.

**Table 2:** Tree of themes and categories from the qualitative analysis.

Themes	Categories			
	Video		Paper	
	Positive Aspects	Negative Aspects	Positive Aspects	Negative Aspects
Aspects related to the format of presentation	More realistic appreciation of occupational performance Non-verbal communication access Appreciation of the environment context	The information is less specific and more difficult to find	Accessibility clarity	Less information
Motivation to learn from the case	Enjoyable			Boring
Professional abilities acquired	Approach of the professional role of occupational therapist Ability to empathize			Does not generate empathy

## Data Availability

Data is contained within the article.

## References

[1] Kamat S.K., Marathe P.A., Patel T.C., Shetty Y.C., Rege N.N. (2012). Introduction of case based teaching to impart rational pharmacotherapy skills in undergraduate medical students. Indian J. Pharmacol..

[2] Kaur G., Rehncy J., Kahal K.S., Singh J., Sharma V., Matreja P.S., Grewal H. (2020). Case-Based Learning as an Effective Tool in Teaching Pharmacology to Undergraduate Medical Students in a Large Group Setting. J. Med. Educ. Curric. Dev..

[3] Mahajan R., Badyal D.K., Gupta P., Singh T. (2016). Cultivating Lifelong Learning Skills During Graduate Medical Training. Indian Pediatr..

[4] McLean S.F. (2016). Case-Based Learning and its Application in Medical and Health-Care Fields: A Review of Worldwide Literature. J. Med. Educ. Curric. Dev..

[5] Thistlethwaite J.E., Davies D., Ekeocha S., Kidd J.M., MacDougall C., Matthews P., Purkis J., Clay D. (2012). The effectiveness of case-based learning in health professional education. A BEME systematic review: BEME Guide No. 23. Med. Teach..

[6] Ghosh S. (2007). Combination of didactic lectures and case-oriented problem-solving tutorials toward better learning: Perceptions of students from a conventional medical curriculum. Adv. Physiol. Educ..

[7] Raza S.A., Qazi W., Umer B. (2019). Examining the impact of case-based learning on student engagement, learning motivation and learning performance among university students. J. Appl. Res. High Educ..

[8] Heinrich C., Pennington R.R., Kuiper R. (2012). Virtual Case Studies in the Classroom Improve Student Knowledge. Clin. Simul. Nurs..

[9] Bennett S., Rodger S., Fitzgerald C., Gibson L. (2017). Simulation in Occupational Therapy Curricula: A literature review. Aust. Occup. Ther. J..

[10] Bradley P. (2006). The history of simulation in medical education and possible future directions. Med. Educ..

[11] Maran N.J., Glavin R.J. (2003). Low- to high-fidelity simulation—A continuum of medical education?. Med. Educ..

[12] Kraft S., Wise H.H., Jacques P.F., Burik J.K. (2013). Discharge planning simulation: Training the interprofessional team for the future workplace. J. Allied. Health.

[13] Brown T., Williams B. (2009). The Use of DVD Simulation as an Interprofessional Education Tool with Undergraduate Occupational Therapy Students. Br. J. Occup. Ther..

[14] Basu Roy R., McMahon G.T. (2012). Video-based cases disrupt deep critical thinking in problem-based learning. Med. Educ..

[15] Chan L.K., Patil N.G., Chen J.Y., Lam J.C.M., Lau C.S., Ip M.S.M. (2010). Advantages of video trigger in problem-based learning. Med. Teach..

[16] Yadav A., Phillips M.M., Lundeberg M.A., Koehler M.J., Hilden K., Dirkin K.H. (2011). If a picture is worth a thousand words is video worth a million? Differences in affective and cognitive processing of video and text cases. J. Comput. High Educ..

[17] de Leng B., Dolmans D., van de Wiel M., Muijtjens A., van der Vleuten C. (2007). How video cases should be used as authentic stimuli in problem-based medical education. Med. Educ..

[18] Ikegami A., Ohira Y., Uehara T., Noda K., Suzuki S., Shikino K., Kajiwara H., Kondo T., Hirota Y., Ikusaka M. (2017). Problem-based learning using patient-simulated videos showing daily life for a comprehensive clinical approach. Int. J. Med. Educ..

[19] Nunohara K., Imafuku R., Saiki T., Bridges S.M., Kawakami C., Tsunekawa K., Niwa M., Fujisaki K., Suzuki Y. (2020). How does video case-based learning influence clinical decision-making by midwifery students?. An exploratory study. BMC Med. Educ..

[20] Woodham L.A., Ellaway R.H., Round J., Vaughan S., Poulton T., Zary N. (2015). Medical Student and Tutor Perceptions of Video Versus Text in an Interactive Online Virtual Patient for Problem-Based Learning: A Pilot Study. J. Med. Internet. Res..

[21] LeeSing A.C., Miles C.A. (1999). The Relative Effectiveness of Audio, Video, and Static Visual Computer-Mediated Presentations. Can. J. Educ..

[22] Reigeluth C.M. (2012). Instructional Theory and Technology for the New Paradigm of Education. Rev. Educ. Distancia RED.

[23] Oosthuizen H. (2019). Speech therapy students’ perceptions of authentic video cases in a theory module on child language disorders. South Afr. J. Commun. Disord..

[24] Keller J.M. (2010). Motivational Design for Learning and Performance: The ARCS Model Approach.

[25] Keller J.M. (2008). First principles of motivation to learn and e3-learning. Distance Educ..

[26] Keller J.M., Suzuki K. (2004). Learner Motivation and E-Learning Design: A Multinationally Validated Process. J. Educ. Media..

[27] Rochmawati E., Wiechula R. (2010). Education strategies to foster health professional students’ clinical reasoning skills. Nurs. Health Sci..

[28] Murphy L., Stav W. (2018). The Impact of Online Video Cases on Clinical Reasoning in Occupational Therapy Education: A Quantitative Analysis. Open J. Occup. Ther..

[29] Facione N., Facione P. (2006). Health Sciences Reasoning Test—Test Manual.

[30] Miralles P.M., Valverde M.Á.T. (2011). Terapia Ocupacional en Salud Mental. Masson. https://dialnet.unirioja.es/servlet/libro?codigo=581524.

[31] Moruno-Miralles P., Reyes-Torres A., Talavera-Valverde M.-Á., Souto-Gómez A.-I., Márquez-Álvarez L.-J. (2020). Learning and Development of Diagnostic Reasoning in Occupational Therapy Undergraduate Students. Occup. Ther. Int..

[32] Rogers J.C., Holm M.B. (1991). Occupational Therapy Diagnostic Reasoning: A Component of Clinical Reasoning. Am. J. Occup. Ther..

[33] Di Serio Á., Ibáñez M.B., Kloos C.D. (2013). Impact of an augmented reality system on students’ motivation for a visual art course. Comput. Educ..

[34] Díaz V.M., Almenara J.C., Pérez O.M.G. (2018). Motivación y realidad aumentada: Alumnos como consumidores y productores de objetos de aprendizaje. Motivation and augmented reality: Students as consumers and producers of learning objects. Aula Abierta.

[35] Loorbach N., Peters O., Karreman J., Steehouder M. (2015). Validation of the Instructional Materials Motivation Survey (IMMS) in a self-directed instructional setting aimed at working with technology. Br. J. Educ. Technol..

[36] American Journal of Occupational Therapy (2014). Occupational Therapy Practice Framework: Domain and Process (3rd Edition). Am. J. Occup. Ther..

[37] Glaser B., Strauss A. (1967). Discovery of Grounded Theory: Strategies for Qualitative Research.

[38] Sampieri R.H., Mendoza C.P. (2018). Metodología de la Investigación. Las rutas Cuantitativa, Cualitativa y Mixta.

[39] Curtin M., Fossey E. (2007). Appraising the trustworthiness of qualitative studies: Guidelines for occupational therapists. Aust. Occup. Ther. J..

[40] Richardson M., Abraham C., Bond R. (2012). Psychological correlates of university students’ academic performance: A systematic review and meta-analysis. Psychol. Bull..

[41] Lu J., Chan L.K. (2015). Differ in Socio-Cognitive Processes? Some Comparisons Between Paper and Video Triggered PBL. Interdiscip. J. Probl.-Based Learn..

[42] Keller J.M. (1979). Motivation and Instructional Design: A Theoretical Perspective. J. Instr. Dev..

[43] Mattingly C., Fleming M.H. (1994). Clinical Reasoning: Forms of Inquiry in a Therapeutic Practice.

[44] Miralles P.M., Valverde M.Á.T., Garlito P.A.C. (2009). Clinical Reasoning in Occupational Therapy. World Fed. Occup. Ther. Bull..

[45] Valverde M.Á.T. (2015). Razonamiento Clínico y Diagnóstico en Terapia Ocupacional.

[46] Klein E.L., Ramos D.S. (2018). Possibilities and challenges of collaborative learning in Higher Education. Educacao.

